# Adenocarcinoma of Mullerian Origin Found Through an Elective Inguinal Hernia Resection: A Case Report

**DOI:** 10.7759/cureus.59929

**Published:** 2024-05-08

**Authors:** Marco Bermudez, Maria Tole, Tabata E Hernandez, Akshay Agrawal, Ivette Vigoda

**Affiliations:** 1 Internal Medicine, St. Barnabas Hospital (SBH) Health System, Bronx, USA; 2 Oncology, St. Barnabas Hospital (SBH) Health System, Bronx, USA

**Keywords:** obgyn oncology, routine histologic exam, incidental cancer, umbilical hernia repair, ovarian adenocarcinoma

## Abstract

We report an asymptomatic 59-year-old female undergoing an elective umbilical hernia excision who was found to have an ovarian adenocarcinoma within the excised hernia. Patients are rarely diagnosed with cancer after an umbilical hernia excision. An excised hernia is rarely the means for an initial diagnosis of cancer. We describe a case of an ovarian carcinoma incidentally found through an umbilical hernia excision with consequential treatment with neoadjuvant platinum-based chemotherapy followed by debulking surgery with a total hysterectomy with bilateral salpingo-oophorectomy with a transoperative pathology report of a high-grade serous carcinoma located in the left fimbrial frond surrounded by a background of serous tubal intraepithelial carcinomas. This case demonstrates the need to perform histological examinations of all excised hernias, even in asymptomatic patients, as malignancy can be found inside a hernia, and it emphasizes the importance of considering adenocarcinomas of Mullerian origin in the differential diagnosis of a malignancy found in a hernia in an asymptomatic female patient.

## Introduction

A hernia containing cancer after routine postoperative histologic examination is an infrequent incidental finding. An electively excised hernia is also an unusual primary way to diagnose occult metastatic intra-abdominal cancers [1]. Therefore, we present a case of a 59-year-old asymptomatic female undergoing an elective umbilical hernia excision, which was found to have an ovarian adenocarcinoma within the hernia after routine histologic examination. This case emphasizes the need for routine histologic examination of all excised hernias, as this can be the first diagnostic evidence of occult cancer in an asymptomatic female patient. This case report was prepared following the CAse REport (CARE) guidelines [2].

## Case presentation

A 58-year-old woman with a medical history of gallstone pancreatitis and laparoscopic cholecystectomy four years prior presented to the surgery clinic with the complaint of an umbilical hernia that had recently started to cause discomfort. The patient reported that the umbilical hernia appeared two weeks after laparoscopic cholecystectomy four years prior. It had always been small in size and had not enlarged recently. She reported three episodes of moderate dull abdominal pain in the epigastric and periumbilical regions in the weeks prior to coming to the clinic. Currently, she denied any constitutional symptoms, including fever, weight loss, anorexia, and night sweats. The patient had no personal or family history of cancer.

**Figure 1:**
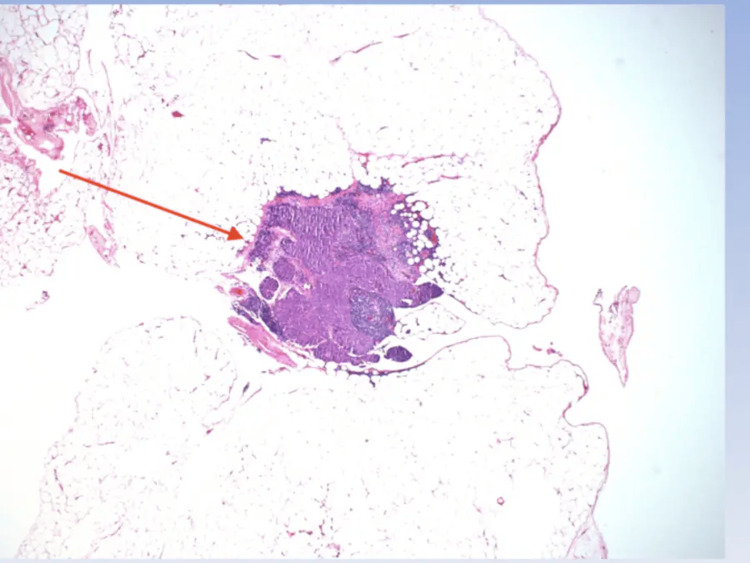
Low power view of adipose tissue within hernia sac with a focus of atypical cells and associated lymphoid tissue (40X).

**Figure 2:**
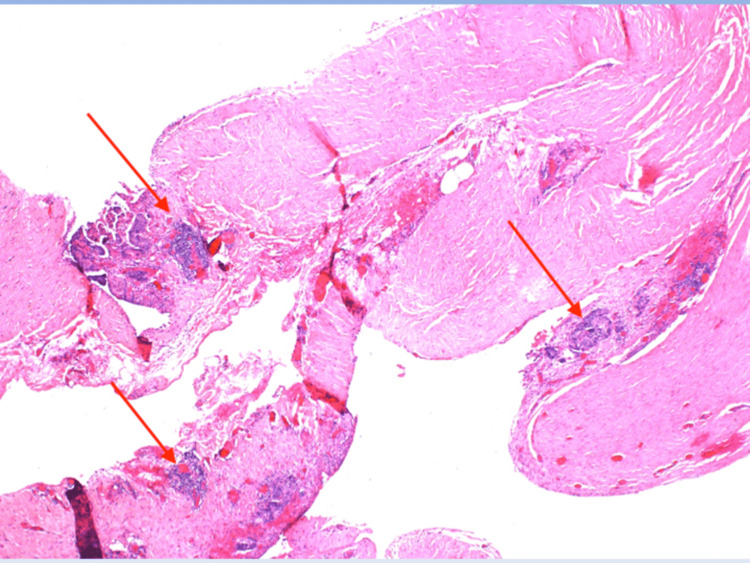
Fragments of hernia sac with scattered foci of atypical cells with papillary features (200X).

**Figure 3:**
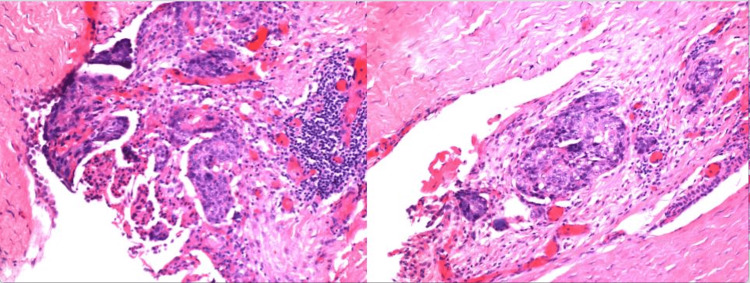
Surface focus of atypical cells with papillary features scattered within fragments of hernia sac (200X).

**Figure 4:**
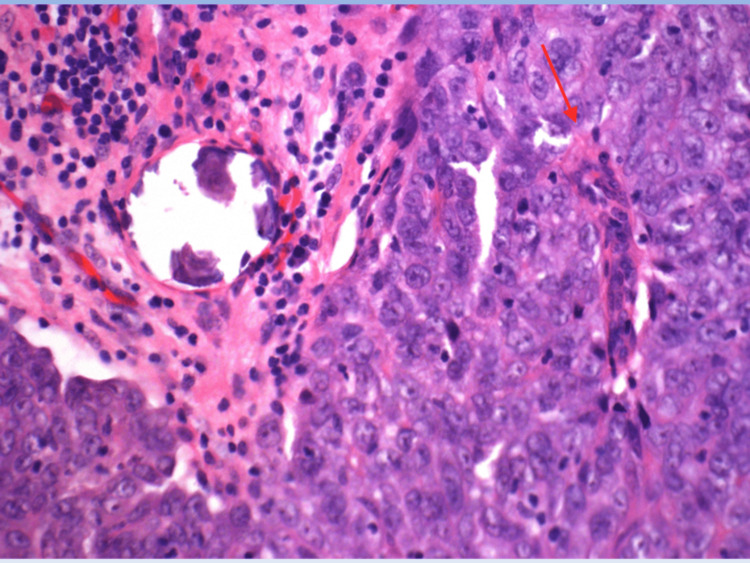
High power view of atypical cells and associated lamellar calcification, consistent with psamomma body (4000X).

The patient underwent umbilical hernioplasty. The hernial sac contents were sent for pathology evaluation. The pathology report was significant for an omentum containing a single focus of metastatic carcinoma with associated lymphoid material with fibrosis in fibro adipose tissue. Immunohistochemical stains for tumor characterization were diffusely positive for Ber-EP4, PAX8, WT-1, p53, p16, ck7, and estrogen receptor (ER). Immunohistochemistry (IHQ) was focally positive for p40. IHQ stains were negative for progesterone receptor (PR), calretin, ck20, and vimentin. IHQ was compatible with metastatic adenocarcinoma, favoring gynecological/ovarian primary. A hernia sac was also positive for fibroconnective tissue with rare foci of metastatic carcinoma (Figures 1-4).

**Figure 5:**
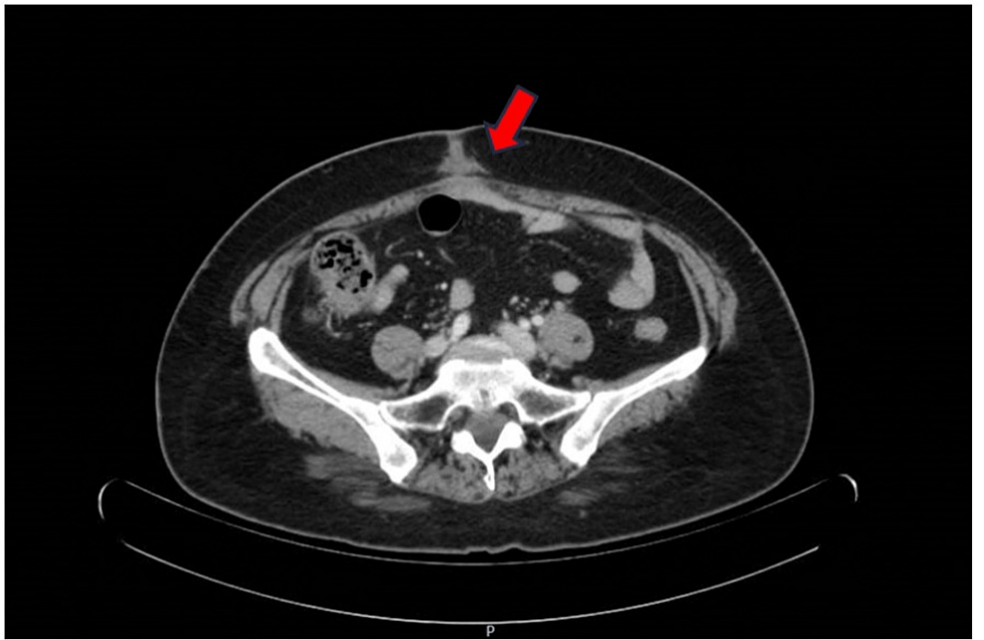
CT scan of the abdomen showing haziness of subcutaneous fat in the periumbilical area, probably sequela of umbilical hernia repair. CT: Computed tomography

Further workup was ordered. A computed tomography (CT) scan with contrast of the chest, abdomen, and pelvis did not demonstrate apparent focal colonic, gastric, uterine, or ovarian masses with no significant retroperitoneal adenopathy. It also reported a cholecystectomy and changes consistent with a recent ventral hernia repair (Figure 5).

**Figure 6:**
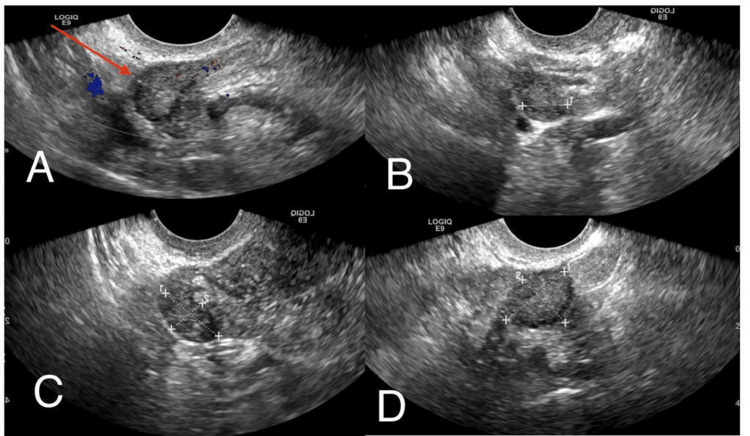
Transvaginal ultrasound showing a retroverted uterus with normal endometrial stripe thickness and a trace amount of free fluid in the endometrial cavity. Unremarkable ovaries. No free pelvic fluid. There is no evidence of ovarian, uterine, or adnexal mass at the moment of diagnosis.

Transvaginal ultrasound demonstrated a retroverted uterus, a normal endometrial stripe thickness, and a trace amount of free fluid in the endometrial cavity with unremarkable ovaries. No free pelvic fluid was demonstrated (Figure 6).

Cancer antigen 125 (CA 125) was found to be normal at 24.7 U/mL. Cancer antigen 19-9 (CA 19-9) was found to be normal at 14. Carcinoembryonic antigen (CEA) was normal, too, at 0.8 ng/mL (Table 1).

**Table 1:** Initial workup WBC: White blood cell count; MCV: Mean corpuscular volume; MCH: Mean corpuscular hemoglobin; ALT: Alanine aminotransferase; AST: Aspartate aminotransferase; CEA: Carcinoembryonic antigen; CA 19-9: Cancer antigen 19-9; CA 125: Cancer antigen 125

Parameters	Value	Normal range
WBC (x10^3^/mm^3^)	5.4	(4.0-10 x10^3^/mm^3^)
Neutrophils	58.5%	(34-71.1%)
Eosinophils	3.0%	(0.7-5.8%)
Monocytes	8.3%	(4.7-12.5%)
Lymphocytes	29.2%	(19.3-51.7%)
Basophils	0.6%	(0.1-1.2%)
Platelets (x10^3^/mm^3^)	328	(150-450 x10^3^/mm^3^)
Hemoglobin (g/dL)	14.5	(11.2-15.7)
MCV (fL)	89.2	(79.4-94.8 fl)
MCH (pg)	30	25.6 - 32.2 pg
Creatinine (mg/dL)	0.7	0.6-1.2
Urea Nitrogen (mg/dL)	11	8-23
ALT IU/L	41	4-36
AST IU/L	37	8-33
ALK phosphatase IU/L	109	38-126
Bilirubin total mg/dL	0.5	0.1-1.2
CEA	0.8	0.0-4.7 ng/dL
CA 19-9	14	0-35 U/mL
CA 125	24.7	0.0-38.1 U/mL

**Figure 7:**
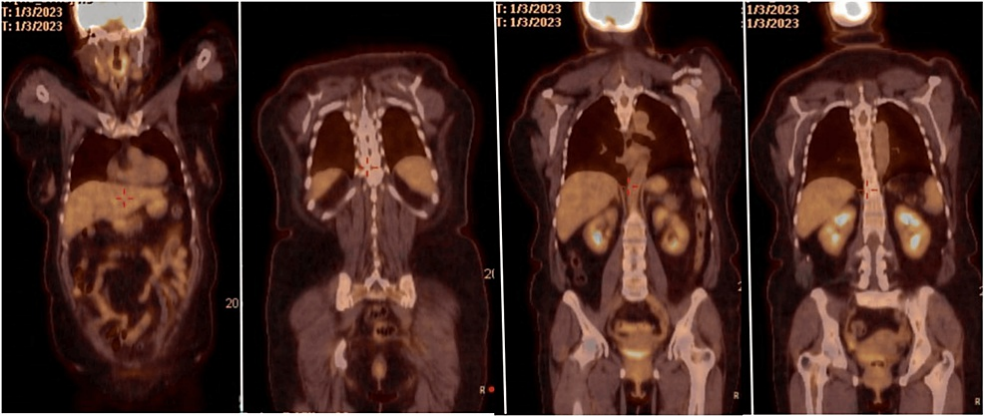
PET/CT skull base to mid-thigh: No suspicious pulmonary masses or nodules are visualized. The liver, adrenals, pancreas, and kidneys appear within normal limits. There is peritoneal thickening posterior to the umbilicus with associated increased metabolic activity. There is no abdominal, pelvic, or inguinal adenopathy or suspicious lymph node metabolic activity. PET/CT: Positron emission tomography/computed tomography

Papanicolau was negative for intraepithelial lesion or malignancy. The human papillomavirus test was negative. Positron emission tomography/computed tomography (PET/CT) showed peritoneal thickening seen posterior to the umbilicus with mild associated increased metabolic activity, standardized uptake value (SUV) max 3.0, findings related to surgical mesh, and postsurgical changes versus residual disease. No definite primary site of adenocarcinoma was identified in this study (Figure 7).

**Figure 8:**
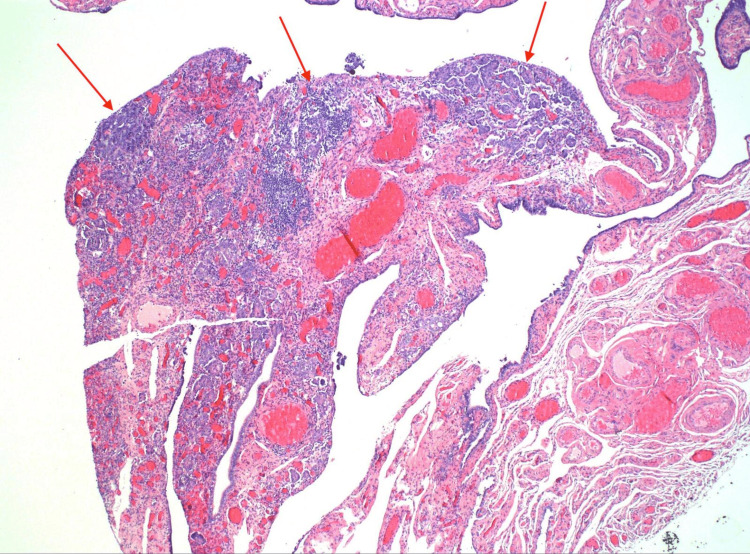
Medium power view of uterine tube stromal involvement by papillary tumor (40X).

The patient was asymptomatic after hernia repair and appeared in good health. Her Eastern Cooperative Oncology Group performance status (ECOG PS) score was zero. A diagnosis of primary peritoneal carcinoma was presumed, consistent with a staging of at least IIIA2 in the International Federation of Gynecology and Obstetrics (FIGO) staging system for ovarian carcinomas. As a result, and to prevent further delay in treatment, the patient received neoadjuvant chemotherapy with three cycles of carboplatin and paclitaxel. CA 125 decreased to 18.3 U/mL. Repeat CT chest, abdomen, and pelvis with contrast was unremarkable for intra-abdominal malignancies, showing changes consistent with ventral hernia repair. Interval debulking surgery was done with a total hysterectomy and bilateral salpingo-oophorectomy, followed by postoperative chemotherapy with an additional three cycles of chemotherapy with carboplatin and paclitaxel. Postoperative surgical pathology showed a high-grade serous carcinoma involving the left uterine tube, invading the fimbrial fond in the background of serous tubal intraepithelial carcinoma (Figure 8).

**Figure 9:**
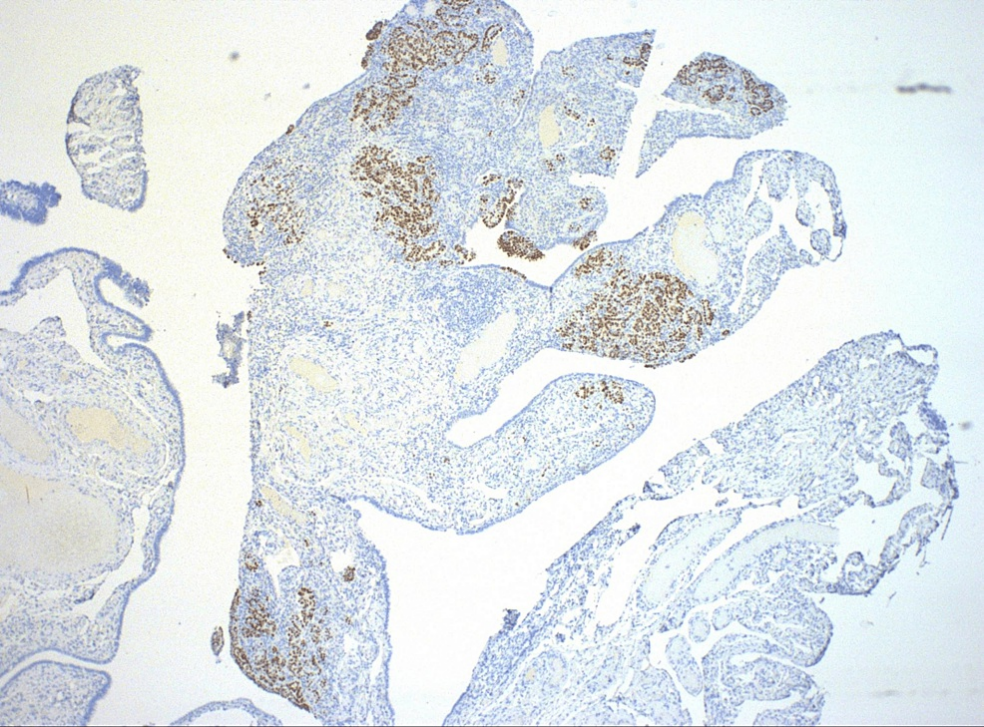
Medium power view of uterine tube with p53 immunostain, highlighting papillary tumor (40X).

**Figure 10:**
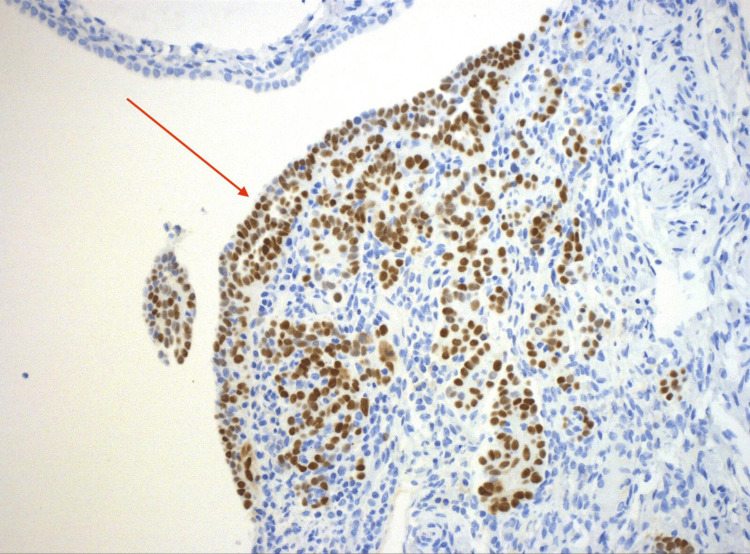
High power view of surface tumor (STIC) with stromal involvement, highlighted by p53 immunostain, supporting a mutation in TP53 (4000X). STIC: Serous tubal intraepithelial carcinoma

Tumor IHQ was diffusely positive for p16, p53, PAX8, and WT-1, with Ki-67 positivity in 40% of cells (Figures 9-10).

The left ovary and right uterine tube had no diagnostic abnormality; the right ovary had a single focus of psammoma body surface implant. There was an inactive endometrium with adenomyosis and a benign cervix in multiple-step sections. A final staging of IIIA2 based on the 2018 FIGO Cancer Report was made.

Repeat imaging four months after surgery with CT chest/abdomen/pelvis (C/A/P) with contrast showed findings consistent with hysterectomy and bilateral salpingo oophorectomies and reactive local pelvic lymph nodes. CA 125 increased to 25.7 U/mL. Germline mutation testing was negative for BRCA1 and BRCA2 gene alterations, deeming the patient not a candidate for poly (ADP-ribose) polymerase (PARP) inhibitor maintenance therapy. The patient is undergoing active surveillance with repeat imaging and serological tests every three months. There is no current evidence of relapse or new metastasis as per imaging, serological, and clinical evidence.

## Discussion

Herein, we reported a rare case of ovarian adenocarcinoma diagnosed through an elective umbilical hernia excision. Elective umbilical hernia excision as a primary means to newly diagnose an occult cancer is an infrequent presentation of the disease in an asymptomatic patient. One of the few studies studying the incidence of metastatic carcinomas found within excised hernias reported a total of 15 patients (0.07%) with metastatic cancer discovered within an excised hernia out of 22,816 hernia specimens evaluated histologically over 39 years in an institution. Of the 15 patients with metastatic carcinoma discovered within the hernias, six patients were newly diagnosed with cancer through a hernia excision, representing a chance of ~0.03% of being newly diagnosed with cancer through an excised hernia. In this study, gastrointestinal adenocarcinomas constituted the majority of primary tumor sites of the cancers found within the hernias. Metastatic ovarian adenocarcinomas were the second most common primary site. Abdominal pain was the symptom most commonly reported preoperatively [3].

Another study addressing the incidence of metastatic carcinomas found through routine pathologic evaluation of excised hernias found an incidence of 0.35% (11/3117) of excised hernias containing metastatic carcinoma. Nine out of 11 patients were women. The mean age of the 11 patients was 67.6 years. Of the 11 patients, three were newly diagnosed with occult cancer through routine histologic evaluation of an excised hernia. Again, the most common symptom preoperatively was abdominal pain. About 5/11 patients had inguinal hernias. The most common primary sites of metastatic carcinomas in this study were from the ovary and fallopian tubes, representing 5/11 cases. Of the 5 cases, 4 were papillary serous carcinomas [1]. Our patient shared similar risk factors, such as post-menopausal age; she was a female, had preoperative symptoms of abdominal pain, and had a papillary serous ovarian adenocarcinoma.

Approximately 225,500 new cases of ovarian cancer are diagnosed each year globally. In the United States, approximately 22,280 new cases of ovarian cancer occur each year, with an annual death toll of 14,240 patients per year [4]. Different risk factors can increase the risk of developing ovarian cancer, such as germline mutations in BRCA1 and BRCA2 DNA repair genes, hormone replacement therapy usage in post-menopausal women, and endometriosis. Ovarian carcinomas can be subdivided into epithelial (90%) and non-epithelial (10%) ovarian cancers. Of the epithelial cancers, high-grade serous carcinoma (HGSC) is the most commonly diagnosed. HGSCs are believed to originate from the distal fallopian tube, from precursor lesions called serous tubal intraepithelial carcinomas (STICs), carrying different gene mutations such as BRCA1, BRCA2, BRIP1, MSH2, MSH6, MLH1, PMS2 and p53 [4].

Unfortunately, no effective screening strategies exist for early detection of ovarian cancer. Genetic screening is offered to any patient diagnosed with invasive ovarian carcinoma alongside family members. Female family members who are found to have a mutation in high-risk genes, such as BRCA1 and BRCA2, are offered risk-reducing bilateral salpingo-oophorectomy at 35-40 years old, with microscopic examination of the ovaries and fallopian tubes following surgery to rule out invasive cancers [4].

The epithelial, serous tumors arising from the ovaries, fallopian tubes, and peritoneum are currently considered a single clinical entity due to their shared behavior, clinical presentation, treatment, and prognosis [5,6]. These tumors can present acutely, subacutely, or constitute an incidental finding during the examination, imaging, or surgery of an asymptomatic patient. At diagnosis, approximately two-thirds of all epithelial ovarian/peritoneal and fallopian tumors are stage III or stage IV, which does not correlate reliably with the presence or severity of symptoms [5,6]. Available clinical data indicates that about 7.2-22.6% of patients with ovarian cancer are asymptomatic at presentation [6].

Retrospective studies demonstrate that the majority of patients with ovarian cancer debuted with symptoms less than six months prior to presentation, all of which are more common in individuals with advanced disease in comparison with those with early-stage cancer [6,7]. Common subacute symptoms are very unspecific, including vague abdominopelvic pain or discomfort (13.5-67%), abdominal swelling and distention (18.5-78%), indigestion (up to 72%), other mild digestive disturbances (19.6-54%), urinary symptoms of frequency or retention (27-45%), menstrual irregularities (11.1-23%), and constitutional symptoms (4.1-57.9%) [6]. Rare presentations comprise rectal bleeding, lymphadenopathy, and symptoms stemming from paraneoplastic syndromes [8].

Patients with advanced disease can also present with acute symptoms requiring urgent management and care, such as abdominal distention due to ascites, shortness of breath secondary to pleural effusion, abdominal obstruction, and other surgical emergencies (e.g., perforation, bleeding, intussusception) [9,10]. Ovarian cancer can debut with venous peripheral thrombosis in about 1% of cases [11,12].

Regarding our clinical case, the patient's only symptom at presentation was dull abdominal pain of a low to moderate intensity that waxed and waned intermittently. This clinical picture is very similar to most patients with advanced-stage ovarian cancer, who exhibit non-specific symptoms that do not correlate with the stage or grade of the underlying malignancy. The duration of symptoms (three months) was also within the normal range of presentation for patients with epithelial ovarian tumors [7]. What is unique about our case is the diagnosis of an underlying malignancy after a hernia excision, as the presence of neoplastic tissue in hernia sacs is rare [13,14].

To the present, ovarian, fallopian, and peritoneal cancer staging is primarily based on surgical findings, which determine the stage of the tumor and the patient's prognosis and also serve as histopathological confirmation of the diagnosis. Imaging is limited to estimating the extent of intra-abdominal and intra-pelvic disease, besides screening for possible distant metastasis [5]. Specific imaging modalities, such as positron emission tomography (PET scan) and magnetic resonance (MR), are mostly reserved for cases of ovarian cancer (at diagnosis or recurrence) in the setting of increasing tumor markers and negative imaging [15,16].

According to the FIGO Cancer Report 2018, if there is a strong suspicion of cancer in a female patient presenting with a pelvic mass, open exploratory laparotomy is indicated. Laparoscopy is more appropriate in cases of suspected benign disease in the setting of negative tumor markers (CA 125, CEA, hCG (human chorionic gonadotropin), AFP (alpha-fetoprotein)) [5].

Prior to surgery, a CT scan of the abdomen and pelvis should be obtained to evaluate the intra-abdominal extent of the malignancy. Also, a chest radiograph should be taken to rule out pleural effusion and overt distal metastasis. No other radiological tests are warranted to rule out extra-abdominal metastasis, as these are uncommon. Elevated levels of tumor markers (especially CA 125) support the diagnosis of cancer with a Mullerian origin [5].

In cases of suspected malignancy, the exploratory laparotomy should proceed with a detailed examination of the abdominopelvic contents. If the primary tumor is limited to the ovary, it should be examined to look for pre or intraoperative capsule rupture. All the peritoneal surfaces must be thoroughly examined and biopsied, especially the suspicious sites. A biopsy from all compromised structures should be obtained, and if possible, the majority of the tumor must be removed in addition to performing total hysterectomy and bilateral salpingo-oophorectomy. The omentum, pelvic, and para-aortic lymph nodes should be removed for histopathological examination [5].

Different blood tests have been proposed for ovarian cancer screening. Unfortunately, they have not been proven effective as a sole strategy for ovarian cancer screening nor have they affected overall survival. One of these is CA 125, which is only increased in 50% of stage I ovarian cancers, lacking sensitivity and specificity, as it is increased in benign disorders, such as ovarian cysts, liver disease, uterine fibroids, and infections. Also, screening strategies combining CA 125 and transvaginal ultrasound have shown unsuccessful results for a reduction in overall mortality for ovarian cancer. Another serum blood test tested has been human epididymis protein 4 (HE4), which has shown better sensitivity and specificity for ovarian cancer compared to CA 125 in various studies. Its clinical significance has been studied, with a recent study in 2018 showing that a combination of CA 125 and HE4 antigen-autoantibody complexes improved the detection of ovarian cancers from 63% using CA 125 alone to 81% using a combination of CA 125 and HE4 antigen-autoantibody complexes [4,14]. In our patient with high-grade ovarian serous adenocarcinoma stage IIIA2, CA 125 levels were never increased, correlating with data showing CA 125 levels may not be elevated in ovarian cancers, lacking sensitivity.

Treatment for newly diagnosed ovarian cancer is based on primary surgical cytoreduction. The stage of the cancer determines the extent of the surgery. Staging is based on FIGO Ovarian Cancer Staging [4,5]. 

In patients with biopsy-proven evidence of pathology consistent with at least adenocarcinoma of Mullerian origin stage IC, II and/or high-grade serous adenocarcinoma of Mullerian origin, systemic chemotherapy is indicated, with a platinum-based regimen combined with taxanes [4,5]. In our patient, as preoperative staging through radiological and pathological studies were consistent with at least stage III ovarian adenocarcinoma, neoadjuvant chemotherapy (NACT) followed by interval debulking surgery and additional chemotherapy post-surgery was performed, as several studies have shown comparable outcomes between first-line surgery with adjuvant chemotherapy compared to NACT followed by surgery and postoperative chemotherapy, with similar outcomes in progression-free survival and overall survival in patients who received NACT. The patient received three cycles of carboplatin and paclitaxel followed by total hysterectomy with bilateral salpingo-oophorectomy plus an additional three cycles of the same mentioned regimen to complete a total of six cycles of chemotherapy. The patient is currently undergoing surveillance with scheduled two-month follow-ups, recurrent CA 125 testing, and surveillance imaging with PET/CT scans. The prognosis of our patient is poor, as more than 80% of advanced-stage ovarian cancers will recur. As our patient was found to have no germline mutations of BRCA1/2, she is currently not a candidate for maintenance olaparib [4,5]. 

## Conclusions

In conclusion, ovarian metastases can be contained within hernias containing peritoneum in asymptomatic female patients with undiagnosed primary ovarian adenocarcinomas. Hernias containing metastatic cancer can be the first and sole sign of hidden carcinomas. Our case demonstrates this sporadic event and demonstrates an argument favoring routine histological examination of all excised hernias, especially in women, as a hidden carcinoma within a hernia can be the first and only sign and evidence of an underlying asymptomatic intra-abdominal malignancy in this population, such as adenocarcinoma of Mullerian origin, as it involves the peritoneum, a frequent site of metastasis.
